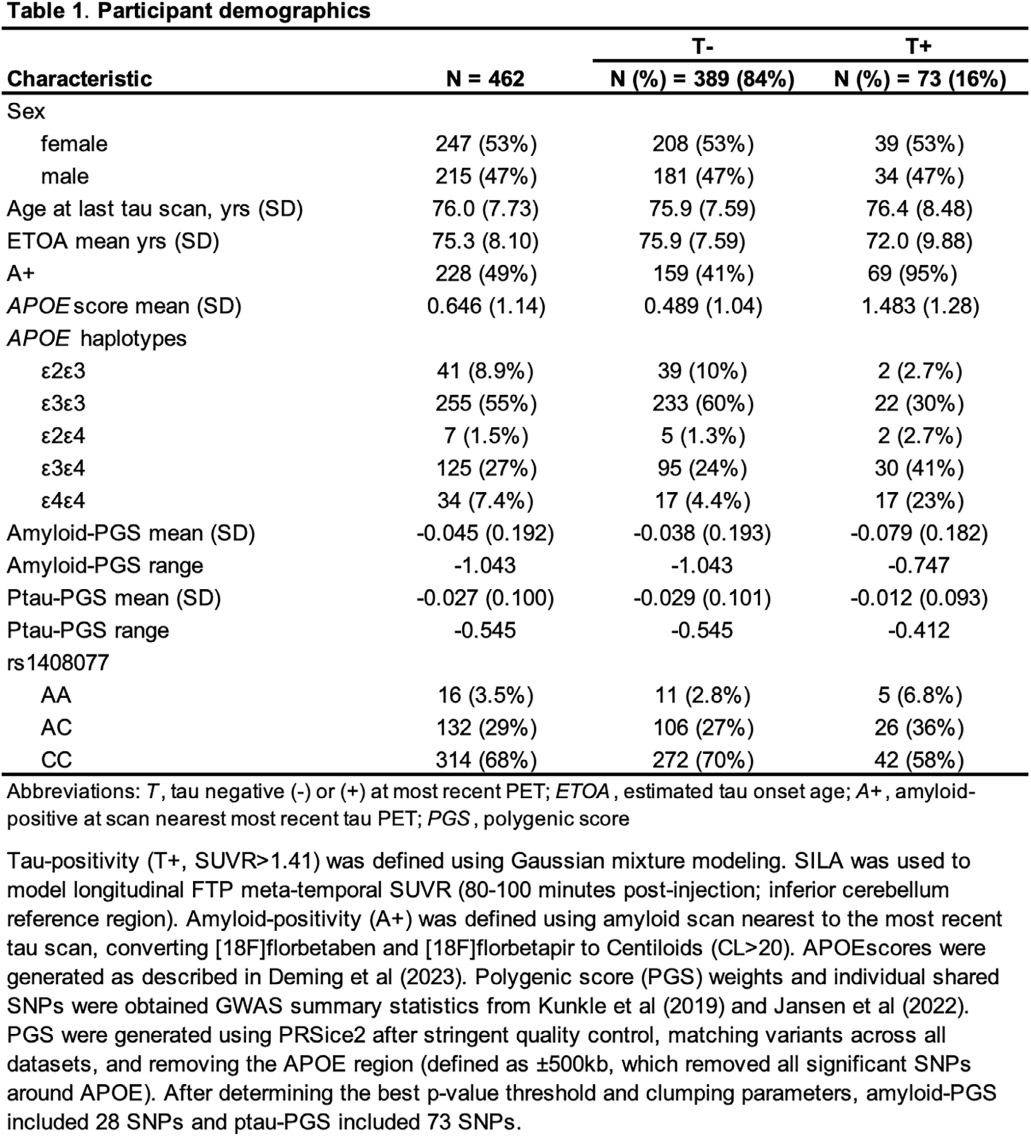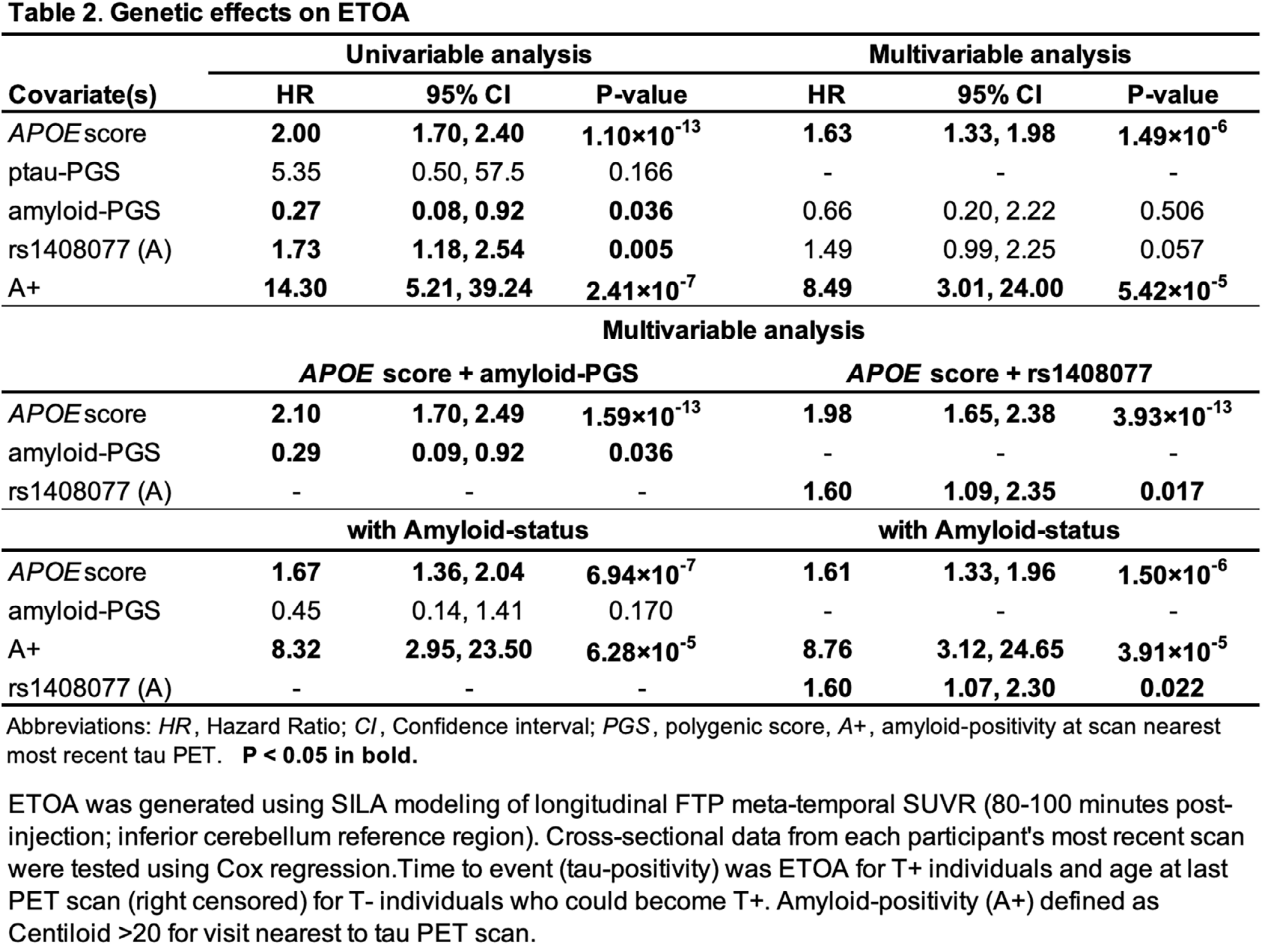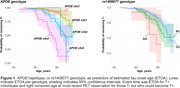# Alzheimer’s disease genetic risk factors’ influence on tau onset age

**DOI:** 10.1002/alz.086038

**Published:** 2025-01-03

**Authors:** Yuetiva Deming, Jordan P Teague, Margo B. Heston, Yazan Hammad, Elena Ruiz De Chavez, Jacob Morse, Richard J Chappell, Rebecca E. Langhough, Megan L. Zuelsdorff, Corinne D. Engelman, Tobey J. Betthauser, Alzheimer’s Disease Neuroimaging Initiative (ADNI)

**Affiliations:** ^1^ School of Medicine & Public Health, University of Wisconsin‐Madison, Madison, WI USA; ^2^ Wisconsin Alzheimer’s Disease Research Center, University of Wisconsin School of Medicine and Public Health, Madison, WI USA; ^3^ Department of Medicine, University of Wisconsin‐Madison School of Medicine and Public Health, Madison, WI USA; ^4^ Wisconsin Alzheimer’s Disease Research Center, University of Wisconsin‐Madison, Madison, WI USA; ^5^ Department of Medicine, University of Wisconsin School of Medicine and Public Health, Madison, WI USA; ^6^ University of Wisconsin ‐ Madison, Madison, WI USA; ^7^ University of Wisconsin‐Madison, Madison, WI USA; ^8^ Wisconsin Alzheimer's Disease Research Center, University of Wisconsin School of Medicine and Public Health, Madison, WI USA; ^9^ Center for Health Disparities Research, Department of Medicine, School of Medicine and Public Health (SMPH), University of Wisconsin‐Madison, Madison, WI USA; ^10^ University of Wisconsin‐Madison, School of Medicine and Public Health, Madison, WI USA; ^11^ Alzheimer’s Disease Research Center, University of Wisconsin School of Medicine and Public Health, Madison, WI USA; ^12^ University of Wisconsin‐Madison School of Medicine and Public Health, Madison, WI USA

## Abstract

**Background:**

New methods developed to estimate when AD biomarkers became abnormal in individuals have shown considerable heterogeneity in amyloid and tau pathology onset age. This work used polygenic scores (PGS) generated from CSF Aβ_42_ and ptau_181_ GWAS, individual‐level genetic data, and estimated tau onset age (ETOA) to identify genetic influences on tau onset beyond *APOE*.

**Method:**

Participants from the Alzheimer’s Disease Neuroimaging Initiative (ADNI) with genetic data, CSF biomarkers (Aβ_42_ and ptau_181_), and longitudinal [^18^F]Flortaucipir (FTP) tau PET were analyzed (N = 462). Sampled iterative local approximation (SILA) was used to model longitudinal FTP meta‐temporal SUVR and generate ETOA for T+ and T‐ participants who could become T+, based on tau‐positivity (T+) defined using Gaussian mixture modeling (SUVR>1.41). Amyloid‐ and ptau‐PGS were generated from publicly available summary data, excluding the *APOE* region (±500kb). *APOE* neuropathology‐based scores (*APOE*score) were used to test *APOE*. We analyzed individually a set of 14 SNPs that were previously associated with AD risk (P<1 × 10^‐5^) and both CSF biomarkers (P<0.05). Amyloid‐PGS, ptau‐PGS, *APOE*score, and individual SNPs were tested as ETOA predictors using Cox regression modeling.

**Result:**

73 (16%) participants were T+ by their last tau PET (Table 1). *APOE*score was significantly associated with earlier ETOA (P = 1.10 × 10^‐13^; Figure 1) as was the amyloid‐PGS (P = 0.036), but ptau‐PGS was not (P = 0.166; Table 2). An intronic variant in *complement receptor 1* (*CR1*), rs1408077 (A allele, MAF_EUR_ = 0.19) which was previously associated with higher AD risk and AD‐associated changes of CSF biomarkers, was also significantly associated with earlier ETOA (P = 0.005; Figure 1). The association remained significant (P = 0.017) after adding the *APOE*score (P = 3.93 × 10^‐13^) and both were still significantly associated with ETOA (P_rs1408077_ = 0.022 and P*_APOE_*_score_ = 1.50 × 10^‐6^) after accounting for amyloid‐positivity (P = 3.91 × 10^‐5^; Table 2).

**Conclusion:**

Our preliminary results support a key role for *CR1* in the age when tau pathology becomes abnormal in AD. The tested variant, rs1408077, is in high linkage disequilibrium with a missense variant, rs2296160, and was previously associated with *CR1* expression in the temporal cortex. This adds to previous studies that reported an interaction between *CR1* and *APOE‐ε4* associated with episodic memory decline. Future work will include genome‐wide analyses of biomarker timing and multi‐trait analyses.